# Security and skills: the two key issues in health worker migration

**DOI:** 10.3402/gha.v7.24194

**Published:** 2014-07-28

**Authors:** Posy Bidwell, Pallavi Laxmikanth, Claire Blacklock, Gail Hayward, Merlin Willcox, Wim Peersman, Shabir Moosa, David Mant

**Affiliations:** 1 Department of Primary Care Health Sciences, University of Oxford, Oxford, UK; 2 Department of Primary Health Care and Family Medicine, Ghent University, Ghent, Belgium; 3 Department of Family Medicine and Primary Health Care, Faculty of Health Sciences, University of Witwatersrand, Johannesburg, South Africa

**Keywords:** health worker, migration, brain drain, insecurity, de-skilling, South Africa

## Abstract

**Background:**

Migration of health workers from Africa continues to undermine the universal provision of quality health care. South Africa is an epicentre for migration – it exports more health workers to high-income countries than any other African country and imports health workers from its lower-income neighbours to fill the gap. Although an inter-governmental agreement in 2003 reduced the very high numbers migrating from South Africa to the United Kingdom, migration continues to other high-income English-speaking countries and few workers seem to return although the financial incentive to work abroad has lessened. A deeper understanding of reasons for migration from South Africa and post-migration experiences is therefore needed to underpin policy which is developed in order to improve retention within source countries and encourage return.

**Methods:**

Semi-structured interviews were conducted with 16 South African doctors and nurses who had migrated to the United Kingdom. Interviews explored factors influencing the decision to migrate and post-migration experiences.

**Results:**

Salary, career progression, and poor working conditions were not major push factors for migration. Many health workers reported that they had previously overcome these issues within the South African healthcare system by migrating to the private sector. Overwhelmingly, the major push factors were insecurity, high levels of crime, and racial tension. Although the wish to work and train in what was perceived to be a first-class care system was a pull factor to migrate to the United Kingdom, many were disappointed by the experience. Instead of obtaining new skills, many (particularly nurses) felt they had become ‘de-skilled’. Many also felt that working conditions and opportunities for them in the UK National Health Service (NHS) compared unfavourably with the private sector in South Africa.

**Conclusions:**

Migration from South Africa seems unlikely to diminish until the major concerns over security, crime, and racial tensions are resolved. However, good working conditions in the private sector in South Africa provide an occupational incentive to return if security did improve. Potential migrants should be made more aware of the risks of losing skills while working abroad that might prejudice return. In addition, re-skilling initiatives should be encouraged.

The worldwide shortage of health workers is estimated to be approximately 4.3 million and countries with the highest burden of disease are those which are worse affected (1). Low- and middle- income countries (LMICs) currently face severe human resources for health (HRH) shortages and this reduces their ability to provide even the most basic health care to their citizens (2). Migration is said to be one of the three major factors exacerbating this situation because the flow of health workers from LMICs to high-income countries is occurring on a major scale (3, 4).

Demand for health workers in many high-income countries has certainly fuelled international migration (5). Some countries have recognised the detrimental consequences of such widespread, unregulated international migration and have developed bilateral agreements to guide recruitment practices. The United Kingdom was a pioneer of such agreements and instigated measures to reduce the number of South African health workers who are newly appointed to work within the country. In 2003, over 3,000 South African doctors registered at the General Medical Council (GMC) for the first time (6). The same year, both countries signed a Memorandum of Understanding (MOU) in order to curb active recruitment of health workers from South Africa to the United Kingdom (7). The MOU did not prohibit individuals applying to work for the National Health Service (NHS); however, it focussed on providing time-limited placements for health workers within approved facilities and developed a mechanism for on-going communication between the two countries. Blacklock et al. reviewed UK policies to address international recruitment of health workers (including the NHS Code of Practice) and concluded that the 99.9% reduction in new registrations of South African doctors at the GMC from 2003 to 2004 was attributable to the MOU (6). In terms of nurses and midwifes, a 45.2% decrease (from 1,674 to 918) in new registrations was seen over the same period (8). Whilst use of registration data has limitations for measuring migration (9), it provides a useful picture of inflows.

It is argued that migration is primarily *demand-led* (10) and the South African-UK MOU illustrates that by curtailing active recruitment it is possible to reduce the flow of health workers between two countries. The decision to migrate is however, extremely complex and there are other factors besides recruitment practices that need to be considered when studying this phenomenon. Migration occurs because it is perceived that a better life and livelihood exist elsewhere. A combination of ‘push’ and ‘pull’ factors lead to the threshold decision to migrate (11). Common pull factors are often the opposite of push factors and tend to focus on pay, working conditions and professional development (12–14). Push and pull factors have been classified as endogenous (within the healthcare system; e.g. remuneration and working conditions) or exogenous (outside the healthcare system; e.g. quality of life and crime) in order to distinguish between issues that are faced (15). Evidence suggests that exogenous have an influential role with regard to international migration (16).

There is limited qualitative research regarding why South African health workers migrate on an international basis and the experiences of those who have migrated. The United Kingdom is not the only country South African health workers migrate to; other English-speaking countries are popular destinations. For instance of the 23,407 South African health workers practicing a medical profession in selected OECD countries 38% were in the United Kingdom, 30% in the United States, 15% in Australia, 10% in Canada and 7% in New Zealand (17). Two qualitative studies suggest that push factors are more influential than pull factors (16, 18) and it is important to gain a deeper understanding of reasons why health workers migrate. In addition, it is important to understand whether the reality of migration to the United Kingdom correlates to the expectations. McGillis Hall et al.’s study entitled *Is the grass any greener* (19) insinuates that information will be provided about whether pull factors are misconceived; however, whilst data presented in the article indicates that career progression is a major pull factor, there is no in-depth information about whether the nurses’ pre-migration expectations were met. Such insight would provide valuable understanding of the issues that health workers face once they have migrated. It could be supposed that a good migration experience creates ‘stay’ factors (e.g. development of new social and cultural bonds) (15) which prevent the health worker from leaving the country which they migrated to. A bad migration experience could mean that ‘stay’ factors are less influential and it is therefore likely that the health worker will again be exposed to push and pull factors which may result in onward migration or even return migration to the county of origin.

In addition, there is limited information about the United Kingdom–South Africa connection. A clearer understanding of migration dynamics and post-migration information exchange is needed between the United Kingdom and South Africa. By exploring the views of health workers about their decision to migrate and the health systems they left behind it may be possible to identify ways to improve the HRH situation in South Africa, particularly within the public healthcare system. In addition this information is important in order to inform the current and possible future roles of the United Kingdom in this partnership. We therefore conducted a qualitative study to determine the experiences of South African trained health workers who have migrated to the United Kingdom. In this paper, we use the findings of this study to focus on 1) Reasons for migration, and 2) The reality of migration. This study is part of the multi-country HURAPRIM Project which aims to develop interventions and policies to address the HRH crisis in Africa.^1^

## Methods

### Study design

Studies involving South African health workers who have migrated to the United Kingdom have been conducted (20, 21), but these have primarily used quantitative methods and questionnaires. Whilst these studies provide a valuable insight into the importance of specific migration factors, use of pre-defined answers means that it is not possible to gain an in-depth understanding of the reasons for, and experiences of migration (22). This study used qualitative content analysis in order to identify individual reasons, views, and opinions which cannot be captured using quantitative methods (23). Only doctors and nurses were identified to participate as opposed to other health worker cadres.

### Participants

The study aimed to compile a heterogeneous sample, based on age, gender, professional training, and length of time resident in the United Kingdom. Participants did not have to be working as a health worker in the United Kingdom; however, the following inclusion criteria were applied:

1. Trained as a doctor or nurse in South Africa.
2. Had some professional experience of working in South Africa.
3. Living in the United Kingdom at the time the interviews took place.

Several recruitment strategies were used. Several UK-based African health worker organisations were contacted in order to identify potential participants. Due to confidentiality, organisations could not provide personal details for individuals but agreed to circulate information about the study and distributed flyers. This strategy generated few participants. Nursing and care homes were contacted in the Oxfordshire region of the United Kingdom in order to identify whether South African health workers were employed there. No South African employees were identified in any of the homes where contact was possible. Despite this some homes agreed to put posters up in order to advertise the study. The most effective recruitment strategy was through informal networks and then snowballing of respondents.

### Data collection

Female researchers (GH, CB and PL) at the Department of Primary Care Health Sciences, University of Oxford established contacts with potential participants. All researchers have experience in qualitative research. Participants were provided with information about the study. Arrangements were made to meet at a place of convenience for the participant in order for the interview to be conducted. Interviews took place at the respondent's home or place of work, or in the researcher's office. Prior to the interview participants were sent detailed information about the study and a consent form, which was completed before the interview began.

Interviews were facilitated through a semi-structured approach. Semi-structured interviews were chosen as they afford the flexibility to change the question order or add questions where necessary. The topic guide was created using the available literature on the migration motives of South African health workers. Interviews took approximately 60 min. All of the interviews were audio recorded and later transcribed verbatim by either the interviewer or another member of the research team. All transcripts were anonymous. Once interviews began, it became apparent that the data was very rich and yielded a great depth of information that had not been obtained before. Participants were recruited until the point whereby all emerging concepts had been explored and no new themes emerged, that is, data saturation had been reached. The research team decided that data saturation was reached after 16 interviews. Interviews were conducted between January and August 2012.

### Data analysis

Data analysis began from the first interview and used qualitative content analysis. The advantages of this approach are that it takes into account the multifaceted characteristics found in qualitative data and for the corroboration of this evidence (24). Inductive content analysis was used as this study was not based on deductive theories (25). Analysis was an ongoing process and interview texts were read several times. This allowed a preliminary understanding of the migration phenomenon to be obtained as well as the identification of key features of the interviews. Interviews were initially sorted into eight content areas, namely: 1) nature of initial training, 2) employment in South Africa, 3) decision to leave South Africa, 4) decision to move to the United Kingdom, 5) employment in the United Kingdom, 6) overall experience in the United Kingdom, 7) overall experience in South Africa, and 8) future plans. Within each content area, statements made by participants (or ‘meaning units’) were labelled using codes. Codes were then aggregated to form sub-categories and abstraction was used to generate two major categories. Initial coding and independent categorisation were done by GH, CB and PL. In addition PB read through all the transcripts and coded them. Category development was discussed within the research team. NVivo (Version 9) was used to support the management of analysis. The study was approved by the Research Ethics Committee of the University of Oxford Ref: MSD/IDREC/C1/2011/96.

## Results

A total of 16 South African health workers were interviewed, of which there were six doctors and 10 nurses (five of whom also had a midwifery qualification). Four (2 doctors and 2 nurses) were working within a different speciality than that they had originally trained for. Three (two doctors and one nurse) were currently not working within the healthcare system, but instead were studying. Participants were predominantly female (*n*=12), white (*n*=11), and had been in the United Kingdom for more than 5 years (*n*=12). Table 1 presents an overview of the characteristics of the participants.

**Table 1:** Characteristics of participants

	*N*
Professional training	
- Doctor	6
- Nurse	10 (*5 also held a midwifery qualification)
Working in the United Kingdom	
- In the same healthcare professional role	9
- In a different speciality within healthcare	4
- Studying	3
Gender	
- Male	4
- Female	12
Age	
- Less than 35 years old	3
- More than 35 years old	13
Ethnicity	
- White	11
- Non-white	5
Lived in the United Kingdom for	
- Less than 5 years	4
- More than 5 years	12
Dependent children	
- Yes	8
- No	8

Results are presented using two major categories 1) The interaction between push and pull factors experienced by South African health workers prior to migration and 2) The challenges faced by South African health workers living and working in the United Kingdom. Within each category, different sub-categories emerged and these are discussed in detail further on.

**1) The interaction between push and pull factors experienced by South African health workers prior to migration**

Most health workers had migrated for a specific purpose. Several participants had family connections with the United Kingdom and this had influenced their decision to go there as opposed to other English speaking countries. Familiarity with the language and culture were important factors.

Table 1 summarises the major push and pull factors that were identified in the study. These have been categorised as factors inside (endogenous) and outside (exogenous) the workplace in order to highlight the differences. Respondents reported that some factors are experienced both inside and outside the workplace. Interestingly many had attempted to address endogenous factors by leaving the public facilities in order to work in the private sector. However, as these health workers still migrated to the United Kingdom, it would appear that overcoming endogenous factors endogenous factors may only be a temporary solution and that other factors are also influential.

- Concern with insecurity and high crime levels

Insecurity and high crime levels were reported to be a major issue both inside and outside the workplace in South Africa. Several participants mentioned incidences of rape or gang violence at their workplace affecting them or their co-workers. All reported incidences of insecurity took place within public facilities, especially in rural locations, and no events were noted to have taken place within private facilities:

My last hospital, when I'd left there was a paediatrician that was raped in the hospital and you know that is just; why should you put yourself through that when you don't have to. (Interview 17, Doctor)

One night we were held up at gunpoint and you know, the police, it was, yeah they came but it, you know, I mean there were so many feuds going on and that. (Interview 4, Nurse)

Whilst no issues with security and crime were reported at work within the private sector, those who worked in the private sector were still exposed to issues of insecurity outside of their workplace. Insecurity was found to be a recurring theme and encountered by all participants, irrespective of where they worked. The concept of moving to a country with a safe environment was paramount:

South Africa is not a particularly safe place to live and again with a family growing up there, it's a totally different life you know. You are, in a sense, a prisoner in your home. (Interview 15, Doctor)

It (crime) is a major issue and we had a major incident in our home and everyone I know …. they've had some sort of a break in and an incident in their home which isn't nice but it's become almost natural. Everyone says, “Well we're just lucky we're not dead!” But you know, living here you realise it's not “lucky you're not dead”, it's, it shouldn't happen. (Interview 3, Nurse)

- Feelings of racial tension within the workplace

Racial tensions were mentioned explicitly by some white participants. Concern was raised over the lack of opportunities for white professionals especially under the current political circumstances in South Africa:

Even though my CV was pretty good I was rejected and told that they were looking for black doctors only for a period to get the number of black doctors up in the country. So the quota systems made me quite angry and so I gave up eventually and decided to go somewhere else. (Interview 13, Doctor)

One of the reasons why we left is because I was being bullied by some of the black nurses there and I was not coping, definitely not coping with that …. I never ever broached the subject of being bullied, because in South Africa you don't do that type of thing. You don't, it's only when I've got over here that I realise, ‘Hang on, I was being bullied. (Interview 9, Nurse)

Issues of racial tension were more acutely felt within the public sector, in particular the rural facilities:

It was very much a racial situation and based on that, that certainly had a strong influence in my decision making because I felt that it would be very difficult to work in a rural setting in southern Africa as a white person. (Interview 11, Doctor)

- The lure of recruitment strategies used by UK agencies

Both doctors and nurses appreciated that their skills were in demand in the United Kingdom. Nurses reported that they had been contacted by matrons and tutors from the United Kingdom and this had influenced their decision to migrate. Active recruitment by medical agencies and advertisements in magazines were found to be extremely influential:

They (the UK) used to advertise a lot in there as well so I think that was another reason why we came. (Interview 5, Nurse)

I was recruited by a [UK] corporate … I'd met one or two of the clinical directors and because they knew about my background … there was an opportunity. (Interview 15, Doctor)

- Opportunities for career development in the United Kingdom

Our study found that it was only the younger doctors who reported that the reason that they had migrated to the United Kingdom was for career development and specifically this was for postgraduate studies. It was felt that the opportunities to learn research skills were greater in the United Kingdom than in South Africa. Interestingly those who had migrated for this reason were the participants who were planning to return to South Africa:

There were better opportunities here academically for me … I would like to go back …. I feel that I have learnt skills here that I can take back. (Interview 17, Doctor)

My studies here [in the UK] are purely functional to get kind of the skills and expertise to go back and work in the [South African] health system. (Interview 18, Doctor)

- The opportunity to gain work experience in a ‘first class world’

Interestingly, whilst private healthcare in South Africa was held in high regard, a mismatch was noted between this care and the context within which it was provided. As one participant succinctly stated, *Private healthcare in South Africa is excellent, it's truly first world care in a third world country* (Interview 15, Doctor). Nurses in particular indicated that they wanted to experience providing this level of care, but in a first world setting:

It was really hard for me to leave, but it was also exciting because I would have loved to just experience working in a first class world. They always say, you know, England, Australia, America are first class and I just wanted to experience that. (Interview 5, Nurse)

I wanted to go and see UK, I thought that it was a country with glass houses and I wanted to see how it is in a developed world, that's how I came here. (Interview 16, Nurse)

- Increased financial reward in the United Kingdom

Remuneration is conspicuous by its absence from the major push and pull factors presented in Table 2. Interestingly, remuneration was not found to be a major factor that influenced decision to migrate. This opinion resonated with both doctors and nurses:

In the NHS hospital my salary is, well, the same as what a sister in a government hospital would be paid. (Interview 1, Nurse)

Sometimes people move for money … recently the government improved pay for doctors in the public sector to the point that it [salary] is not really becoming such a good reason for people to leave. (Interview 17, Doctor).

**Table 2:** Emerging themes which influenced the decisions of South African health workers to migrate to the United Kingdom

	Reasons to leave South Africa	Reasons to migrate to the United Kingdom
Endogenous (within the health system)	High HIV prevalence Staff shortages Heavy workload Lack of resources Bullied at work Impact of affirmative action on job prospects	Desire to experience a ‘first world healthcare system’ Gain skills that can be used to help the South African system (doctors). Historical active recruitment practices
Exogenous (beyond the health system)	High crime rates Fears over personal safety Concern over political climate (anti-white sentiments) Despondency over state of non-health facilities in the country Corruption	Low crime rates English speaking Historical links Geographically close to South Africa (relative to other English-speaking countries) Close to Europe – increased exposure to travel opportunities Friends/family already living in the United Kingdom Friends/family applying to work in the United Kingdom Personal development Personal development

However, some doctors in this study reported that the major drawback to working in the private sector in South Africa was the difficulty in obtaining reimbursement from medical insurance companies:

The private systems have their faults as well, I mean the medical aid system in South Africa is problematic. Doctors are late in getting paid and they struggle to get their money from the medical aid so you know, not everyone wants to deal with that. (Interview 8, Doctor)

**2) The challenges faced by South African health workers living and working in the United Kingdom**

While there were positive views about life in the United Kingdom, particularly the relief from crime and better security, the reality of migration identified major challenges. In particular, study participants highlighted differences between the working environments within the two countries. Many spoke of their enjoyment of working in South Africa. It is important to note that whilst some participants highlighted the strengths of the South African private healthcare system, they were quick to appreciate that it is not possible to compare it with the NHS:

I think the private healthcare system in South Africa's a little bit better than the UK system. Having said that, I can't really compare it because the UK system is a government funded system. (Interview 2, Nurse)

I did find it [the NHS] colder and I think it is … because in South Africa every patient is a customer. Everyone you see is paying towards your expensive car and whatever so you; patients are kind of gold. (Interview 18, Doctor)

Participants appeared to be philosophical about differences in working practices that they experienced in the United Kingdom compared to South Africa. However, two major themes emerged as negative aspects of migration, namely clinical de-skilling and the associated challenges of working in a different country.

- Mixed feelings about utilisation of skills

Gaining skills, particularly in research, was mentioned by some doctors as a reason for coming to the United Kingdom. However, nurses did not report that gaining skills had been a major motivating factor for migration. Whilst some nurses reported that they had gained skills whilst they had been in the United Kingdom: *I learnt big things like meds management … that was not part of my work over there [South Africa]* (Interview 9, Nurse), the majority felt that since migrating they were actually losing their clinical skills. Nurses reported that in South Africa they were afforded high levels of independence and were able to conduct procedures which they were not allowed to perform in the United Kingdom. In addition nurses felt that they were allowed more authority in the South African workplace and in addition to their clinical duties they were able to contribute to improving policies. Many nurses reported that the amount and level of work given to them in the United Kingdom was described by some as ‘things minor nurses can do’. Nurses firmly believed that they had become so de-skilled whilst working in the United Kingdom that they would find it difficult to go back and work in South Africa:

In the UK you know, putting up a drip, I haven't done that in a long, long time. So I felt slightly like, if you like, deskilled to my peers and because I had to go back; I went into a private hospital to work in a general ward. Just putting up a drip, I couldn't, I didn't have the confidence to do that. (Interview 1, Nurse)

In UK … you are always dependent, you are being told, we are like a remote control, we have got no input in the policies, you have got nothing. In South Africa the policies, they depend on nurses who are able to change policies, to formulate policies, to evaluate policies – something we don't do in England. (Interview 16, Nurse)

- Challenges associated with working in a ‘foreign’ country

While working in the United Kingdom, some participants felt like they were not treated the same as the other nurses, and were treated as ‘foreign’:

In UK if you are a foreigner, you're a foreigner, it's difficult for you to move …. When I came here I was much experienced but if you're a foreigner there isn't much for you and they don't tell you what is expected of you. (Interview 16, Nurse)

Appreciation for the work done, and job satisfaction was a concern of many. Health workers reported that they felt ‘needed’ in South Africa where they were more appreciated and acknowledged for the work they did. The nurses felt that even with the limited resources available in South Africa, the importance of their role as health workers was very high:

What I do find in England is, yes, you find patients that is happy about the treatment you give them and, you know, they will say thank you etc. etc. Whereas South Africa's a different feeling – people really, really, you know, appreciate what you're doing for them. (Interview 5, Nurse)

## Discussion

Content analysis allowed the generation of categories in order to generate knowledge about the phenomenon of South African health worker migration to the United Kingdom. Our findings offer a significant addition to the understanding of the reasons why South African health workers migrated to the United Kingdom and their experiences of this migration. Push and pull factors are clearly evident in this study. Whilst many are context specific, it is clear that both source and destination countries have key responsibilities to mitigate the negative consequences of international health worker migration. Current migration strategies have focussed on bilateral agreements, such as the UK-South African MOU; however, despite the success of some initiatives, international migration of South African health workers continues. Indeed, by 2006, South Africa was the African country with the highest number of doctors abroad, totalling 12,136 (equivalent to one third of its national medical workforce) (2). South African health worker migration has therefore become a major international policy issue (26).

South Africa has been proactive in trying to address HRH issues through the implementation of strategies to lessen pull factors (through the UK MOU) whilst addressing push factors within the country. Within the public sector, a variety of financial and non-financial initiatives (such as the Hospital Revitalisation Programme, Rural and Scarce Skills Allowance, and Occupation Specific Dispensation) have been implemented in order to improve issues of motivation and retention. These incentives have had varying degrees of success at improving retention of health workers within their posts (27, 28). In addition, initiatives such as the ‘Home Coming Revolution’ and the African Health Initiative are working to stimulate the return of South African expatriates.

This study highlights two key issues: 1) insecurity and racial tension in the workplace is a major push factor, and 2) clinical deskilling of health workers has implications for both the individual and the health system within which they work, or could potentially work. These issues have implications for HRH policies in both South Africa and the United Kingdom.

**1) Insecurity and racial tensions at work as a major push factor**

Financial reasons have previously been cited as the major factor for South African health workers to migrate (29). However, this study found that remuneration is no longer as influential as it once was. Factors, such as insecurity and racial tension were more influential in the decision to emigrate. Many of the issues relating to endogenous factors were only found in the public sector. Consistent with previous findings (30), health workers in this study had overcome these push factors by gaining employment within the private sector. South Africa, like many other countries that experience major international emigration, also faces widespread internal migration. This has created a substantial mal-distribution of personnel between rural and urban areas and between public and private facilities; in addition to an overarching move from primary to tertiary healthcare (30–32). Public facilities are worse affected and suffer from a lack of coordination and funding (33). As the majority of the South African population rely on the understaffed public health system (34), it is essential that strategies are found to retain and motivate the health workers employed there.

Working within the private sector was not problem-free and doctors in particular reported that there were issues regarding reimbursement by medical insurance companies. Whilst this was not found to be a major motivating factor for migration and relates only to work in the private healthcare system, it is a theme which has not been identified before. Further research is needed to determine just how influential this factor is and what strategies can be implemented to address it.

A key issue is insecurity at work and health workers need to feel they are safe whilst they are working. Workplace violence is not confined to South Africa; for instance recent studies have shown that it occurs in Jordan (35), Pakistan (36), and China (37). Guidelines have been developed by the World Health Organization (WHO) in order to address workplace violence (38) and governments must implement strategies to ensure occupational safety. Our results suggest if issues of insecurity and racial tension at work were addressed this may attract South Africans back.

**2) Clinical deskilling of migrant health workers**

This study highlights many positive aspects of working in South Africa, for instance, independence, enhanced clinical skills, and a feeling of making a difference. By advertising the benefits of working within South Africa, it is possible that push factors will be less influential. In addition, attention could be drawn to the reality of migration, that is, that pull factors are often misconceptions. Haour-Knipe and Davies (39) propose that migration policies should focus on decreasing factors which made people migrate in the first instance (39) and perhaps if governments played to the strengths of working within the home country this may be incentive enough for many to remain.

Previous studies have highlighted that individuals migrate for better professional opportunities and for skill development (13, 40). The reality seems to be quite the opposite. Whilst our results show that the doctors who migrated for postgraduate studies have gained skills, many of the nurses reported that because their employment opportunities were restricted in the United Kingdom they were becoming ‘deskilled’.

Deskilling is detrimental for all actors involved in healthcare systems – it is psychologically damaging for the health workers and means that neither destination nor source country are fully utilising the talents of the workforce they have trained or employed (41). Deskilling of migrant nurses (42) and doctors (43) has been identified in other studies. For example, South African doctors working in Canada have spoken of their frustration at not being able to use their clinical skills: *The scope of work is limited. I can't do C-sections or give anaesthetics although I had 14 years’ experience* (44). Destination countries, such as the United Kingdom, should explore strategies for using, maintaining, and improving the clinical skills of the migrant health workers who come to work there. For instance, in the United Kingdom, South African nurses could be encouraged to apply for advanced nurse practitioner positions which would allow them to extend and expand their scope of practice. It would be interesting to evaluate whether this would increase their motivation and their contribution to the health system where they are working. In addition, ‘return-to-practice’ programmes for health workers could be extremely beneficial to ensure that returning migrants are refreshed with the skills that they need to work in their home countries. ‘Return to practice’ schemes have been used in the United Kingdom to support nurses and midwives who have had a period away from work and to equip them with the skills that they need to practice under current conditions (45). Such schemes have seen thousands of nurses return to work in the United Kingdom each year (46), and are undoubtedly more cost-effective than training new health workers. Suggestions have been made that destination countries compensate source countries for the loss that they have incurred when health workers migrate (11). Whilst it is difficult to calculate what adequate compensation would be, it could be that destination countries, such as the United Kingdom, provide assistance to ‘return to practice’ schemes within source countries such as South Africa. Such a scheme could be piloted in order to evaluate its effectiveness at encouraging health workers to return to work in South Africa.

There is room for a strong UK-South African collaboration to develop a forum in the United Kingdom. Such a forum could provide information about changes in South Africa, for instance the National Health Insurance and Primary Health Care re-engineering including efforts at improving workplace security and racial harmony. In addition, opportunities could be promoted for useful exchanges between the two countries. The work being done by Homecoming Revolution (http://homecomingrevolution.com/) and African Health Placements (http://www.ahp.org.za/) to attract expatriate health professionals back to South Africa is worth enhancing in such collaboration.

### Study limitations

It was anticipated that it might be difficult to recruit individuals that fulfil certain categories of the sampling frame and efforts were made to ensure that participants were as representative of South African migrant health workers as possible. It was not possible to identify health workers who are employed within the UK health sector and recruitment activities focussed on a specific county within the United Kingdom. There was a low ethnic diversity of participants with mostly white professionals interviewed. Bhorat et al. (47) found in their study of labour migration from Southern Africa that white professionals are more inclined to migrate than other ethnic groups, (47) and our sample could reflect this finding. There is an overrepresentation of respondents who have lived in the United Kingdom for more than 5 years and whilst this could reflect the reduced numbers of South African health workers migrating to the United Kingdom, it means that results cannot be generalised and they are specific to this population.

Whilst the sample size is small, the results provide a valuable insight into the reasons why South African health workers migrate. The results, in particular factors influencing the decision to migrate from South Africa, are likely to bear relevance to South African health workers who have migrated to other English-speaking countries. The results, however, do not claim to be representative of health workers from other African countries and continued qualitative research needs to be done in order to understand health worker migration in a broader sense. In addition, a large-scale quantitative study needs to be done in order to provide numerical measurements of the migration phenomenon and to determine any associations.

## Conclusion

This study sought to understand reasons why South African health workers migrate to the United Kingdom and to gain an understanding of their experiences of this. Previously there had been very limited qualitative information about this phenomenon. This study indicates that factors such as insecurity and racial tension can be extremely influential in the migration process. This insinuates that migration is unlikely to diminish until the major factors over security, crime and racial tensions are resolved. However, good working conditions in the private sector in South Africa provide an occupational incentive to return if security did improve. The themes elucidated in this study indicate that strategies to tackle migration need to work both inside and outside the healthcare system. Furthermore, the results suggest that migrants need to be aware of the realities of migration, in particular potential de-skilling and the impact that this may have on their ability to return to work in their home country if they want to.

## Footnotes

1 http://www.huraprim-project.eu.
